# Rickets Types and Treatment with Vitamin D and Analogues

**DOI:** 10.3390/nu16030416

**Published:** 2024-01-31

**Authors:** Giacomo Biasucci, Valentina Donini, Giuseppe Cannalire

**Affiliations:** 1 Pediatrics and Neonatology Unit, University of Parma, Gugliemo da Saliceto Hospital, 29121 Piacenza, Italy; g.cannalire@ausl.pc.it; 2 Department of Medicine and Surgery, University of Parma, 43121 Parma, Italy; 3 Unit of Pediatrics, Department of Medicine and Surgery, University of Parma, 43121 Parma, Italy; valentinadonini94@gmail.com

**Keywords:** rickets, ergocalciferol, cholecalciferol, FGF23, Burosumab

## Abstract

The definition of “Vitamin D” encompasses a group of fat-soluble steroid compounds of different origins with similar chemical structures and the same biological effects. Vitamin D deficiency and/or a defect in the process of its synthesis or transport predispose individuals to several types of rickets. In addition to cholecalciferol, ergocalciferol, and vitamins D3 and D2, there are also active metabolites for the treatment of this condition which are commercially available. Calcitriol and aphacalcidiol are active metabolites that do not require the renal activation step, which is required with calcifediol, or hepatic activation. The purpose of this review is to summarize current approaches to the treatment of rickets for generalist physicians, focusing on the best vitamin D form to be used in each type, or, in the case of X-linked hypophosphatemic rickets (XLH), on both conventional and innovative monoclonal antibody treatments.

## 1. Introduction

Rickets is a developmental bone disease characterized by reduced or absent endochondral calcification of growth cartilage, resulting in deformation and reduced mineralization of newly formed bone tissue. Rickets classification is mainly based on mineral deficiency and is conventionally defined as calcipenic or phosphopenic [1]. The clinical presentation is heterogeneous and depends on the age of onset and pathogenesis; indications may include arch deformities of the legs, short stature, and joint enlargement. The disorder can be caused by nutritional deficiencies or genetic defects. Although prevalence has decreased in the last two centuries worldwide, rickets remains an important public health concern and a preventable cause of global morbidity and even mortality, especially in developing countries [2]. Prevention is only possible for nutritional rickets, i.e., through dietary supplementation with vitamin D and calcium or enrichment with calcium and vitamin D, alone or in combination with exposure to sunlight. In children aged between 0 and 6 months and 6 and 12 months, the required calcium intake is 200 and 260 mg/day, respectively. In children over 1 year of age, an adequate dose of calcium is 500 mg/day. In addition, all infants should receive 400 IU/day of vitamin D supplementation throughout the first year of life, regardless of their feeding mode. Thereafter, children with a history of symptomatic vitamin D deficiency and those with risk factors that may reduce the synthesis or intake of vitamin D are candidates for supplementation [3]. However, in the absence of these predisposing conditions, children not receiving vitamin D supplementation are not obvious candidates for suffering from nutritional rickets. The aim of this paper is to summarize and highlight, while including information for generalist physicians, current knowledge on the role of vitamin D and its metabolites, as well as that of new monoclonal therapies, in the treatment of different types of rickets. 

## 2. Materials and Methods

A non-systematic review of the literature was performed to search for and select the most up-to-date papers on rickets classification and treatment recommendations worldwide. The terms “rickets” and “classification”, “ergocalciferol”, “cholecalciferol”, “FGF23”, “Burosumab”, “osteoporosis”, “bone health”, and “rickets” and “treatment” were searched for in papers published on PubMed from 2003 to 2023 using the MeSH database. Only reviews, consensus documents, guidelines, recommendations on conventional treatments, and/or RCTs or phase 3 trials focusing on novel therapies were considered; additionally, only papers written in English were selected. A total of 44 papers were chosen and are referred to in this manuscript.

## 3. Vitamin D

The two main forms of vitamin D are ergocalciferol (vitamin D2), which is synthesized by the irradiation of ergosterol in yeast and fungi and then ingested by humans through diet, and cholecalciferol (vitamin D3) generated from 7-dehydrocholesterol, which is present in the plasma membrane of keratinocytes by means of skin exposure to ultraviolet irradiation. Dietary vitamin D2 begins its absorption cycle in the stomach, where pepsin breaks down the protein fraction, and continues in the duodenum, where other digestive enzymes further contribute to the release of vitamin D from the food matrix. Moreover, bile acids initiate mixed micelles containing fat-soluble substances for emulsification and synthesis; these molecules are then absorbed by enterocytes to be firstly released into the lymphatic circulation and then into the blood stream. With regard to vitamin D3 absorption, it is firstly delivered from the keratinocyte plasma membrane to the extracellular space and then into the systemic circulation. Like steroid hormones, vitamin D is transported in the blood stream by being bound to vitamin D-binding protein (vDBP), which acts as a regulator of total and free circulating vitamin D metabolite concentrations.

Both dietary and endogenous vitamin D must be activated. The first step is the hepatic conversion of both vitamins D2 and D3 into their 25-hydroxylated form, calcifediol (25(OH)D2 and D3, respectively), which is carried out by liver microsomal cytochrome P450 2R1 (CYP2R1). The second step of vitamin D activation is the renal conversion of 25(OH)D2 and D3 into their biologically active form, calcitriol (1,25(OH)2D2 and D3, respectively), by kidney CYP27B1. The effects of vitamin D are mediated by a nuclear VDR receptor, which, after entering the cell, heterodimerizes with the retinoic acid X-receptor and interacts with specific DNA sequences known as vitamin D response elements (VDREs); then, it may activate or repress DNA transcription, which is crucial for bone metabolism and calcium uptake [4].

The key function of vitamin D is to optimize intestinal absorption of calcium and phosphorus for proper formation of the bone mineral matrix [5]. Serum 25(OH)D is a negative acute-phase reactant which has implications for acute and chronic inflammatory diseases; it is an unreliable biomarker of vitamin D status after an acute inflammatory insult. Hypovitaminosis D may be the consequence rather than the cause of chronic inflammatory diseases [6].

**Figure 1:**
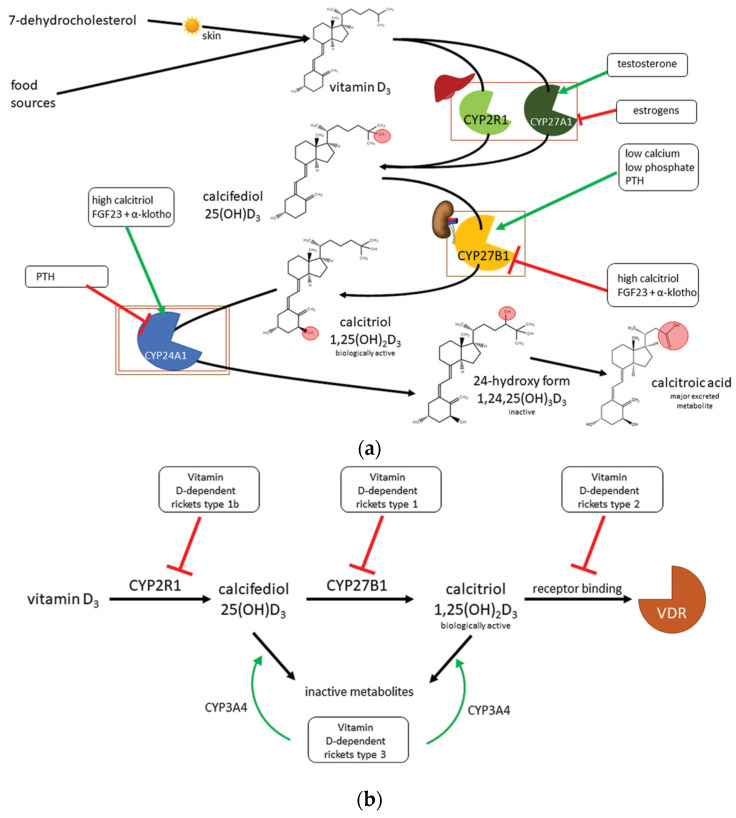
Vitamin D metabolism (**a**) and related genetic disorders (**b**), modified from [4].

Renal calcitriol synthesis is crucial for mineral homeostasis as well as for bone metabolism; this process is tightly regulated in humans. Indeed, calcitriol may inhibit CYP27B1, whereas parathyroid hormone (PTH), whose activity is stimulated by hypocalcemia, activates it, resulting in increased calcitriol production. Nevertheless, in a form of negative feedback, calcitriol suppresses PTH release by upregulating calcium-sensitive receptors and increasing serum calcium concentrations. Direct inhibition of PTH release by the calcitriol/VDR complex has also been demonstrated. The other regulatory signaling pathway is due to fibroblast growth factor 23 (FGF23), which is synthesized by osteoblasts and osteocytes; in this pathway, vitamin D metabolism is modulated based on serum phosphate concentrations. In cases of hyperphosphatemia, FGF23 and its cofactor α-khloto stimulate kidney phosphate excretion, inhibiting CYP27B1 and increasing CYP24A1 expression, thus lowering serum calcitriol concentrations. In turn, calcitriol promotes FGF23 expression (Figure 1) [4].

## 4. Vitamin D Deficiency

Vitamin D deficiency affects more than one billion people worldwide. There has probably been an excessive use of vitamin D testing, which has resulted in an apparent increase in “deficient” children, while diagnosis rates of rickets are only slightly increasing. This slight increase could be attributable to several reasons, including population migration, poverty, and reduced sunlight exposure due to lifestyle factors. Although 1,25(OH)2D is the active form, 25(OH)D is the generally accepted indicator of vitamin D status. To date, circulating 1,25(OH)2D concentrations have received relatively little attention (except in patients with chronic kidney disease) due to its short half-life and significantly lower concentration when compared to circulating 25(OH) vitamin D.

Furthermore, immunological tests of vitamin D2 (derived from supplements and food sources) concentrations may suffer from interference biases. Another confounding factor is that most vitamin D supplements contain ergocalciferol and not cholecalciferol. From analytical point of view, this may affect laboratory results, as immunoassays tend to detect cholecalciferol better than ergocalciferol, thus possibly over-estimating hypovitaminosis D conditions.

When assessed, deficiency rickets, either due to nutritional deficit or to insufficient sunlight exposure, may be associated with many chronic and acute diseases, including childhood dental caries, pre-eclampsia, cancer, type 2 diabetes, autoimmune diseases, cardiovascular diseases, and neurological disorders [7]. As such, this form of rickets representing a major public health concern.

Vitamin D status is usually defined as follows: deficiency, with a serum value lower than 20 ng/mL (<50 nmol/L); suboptimal concentration, with a serum value between 20–30 ng/mL (50–75 nmol/L); and optimal concentration, with a serum value between 30–50 ng/mL (75–125 nmol/L).

Vitamin D deficiency in the general population can be prevented by cholecalciferol supplementation, which should be customized according to age, body weight, sun exposure, eating habits, and lifestyle (Table 1) [8].

Cholecalciferol is the preferred form of vitamin D supplementation at any age. From 11 years of age, if a good rise in serum vitamin D concentrations is not achieved with cholecalciferol, calcifediol may be used, with the serum vitamin D dosage being checked 6–8 days after starting supplementation. For children under 10 years of age, calcifediol supplementation is not recommended. In people aged from 4 to 65 years of age, vitamin D supplementation is only recommended in those who do not have sufficient exposure to sunlight. However, from the age of 65, there is a reduction in the effectiveness of vitamin D skin synthesis, so year-round supplementation is recommended in all individuals [8,9,10].

Available epidemiological data in Italy show a high prevalence of hypovitaminosis D during the entire pediatric age, but mostly in adolescence. It has emerged that the vitamin D status of newborns is influenced by maternal ethnicity and prophylaxis during pregnancy, while the season in which vitamin D testing is carried out, ethnicity, BMI, and sunlight exposure are major determinants in infants and children. The Italian national consensus document, issued by the main Italian pediatric scientific societies, highlights the extra-skeletal functions of vitamin D, underlining its beneficial role not only for bone health but also in the prevention of non-skeletal diseases. The recommendations reinforce the need to ensure adequate circulating vitamin D concentrations throughout the pediatric age. Hence, vitamin D supplementation is recommended for all newborns in the first year of life, regardless of the type of diet and, subsequently, based on the presence of risk factors [11].

## 5. Types of Rickets

Rickets classification is usually defined as calcipenic or phosphopenic, based on the main mineral deficiency [1].

Calcipenic rickets is mainly due to a lack of calcium, combined with low vitamin D availability or function. Therefore, calcipenic rickets may be due to reduced calcium intake or, more frequently, hypovitaminosis D secondary to insufficient sun exposure, low intake, or malabsorption.

Nutritional rickets is the most widespread form of rickets in the world, with an increasing incidence even in Western countries. Vitamin D deficiency is a pandemic in Europe, especially in winter, with a much higher incidence in dark-skinned subjects [8,9].

Vitamin D-dependent genetic rickets is due to a mutation in genes coding for the enzymes involved in vitamin D activation steps and is mainly characterized by hypocalcemia and secondary hyperparathyroidism. Vitamin D-dependent rickets type 1A is due to deficiency in renal 1,α-hydroxylase synthesis, whereas vitamin D-dependent rickets type 1B is caused by hepatic 25-hydroxylase deficiency. Vitamin D-dependent rickets type 2 is characterized by a peripheral resistance to 1,25(OH)_2_D; these forms might be caused by abnormalities in the vitamin D receptor (VDR) (type 2A) or impaired vitamin D receptor function (type 2B). Both are characterized by extremely high circulating 1,25(OH)_2_D concentrations (3–30-fold higher than the norm) [10]. Type 2 may be associated with the presence of alopecia in about 50% of patients. These patients commonly display more severe typical clinical features compared to those who do not have alopecia [12]. Recently, a new form of vitamin D-dependent rickets (type 3), characterized by increased vitamin D degradation, has been discovered. Unlike the recessively inherited type 1 and type 2, this form is transmitted as an autosomal dominant character and may be identified by an activating mutation in the gene coding for P450-dependent enzyme CYP3A4. This enzyme is involved in the hepatic inactivation of some compounds, including the two most important vitamin D metabolites, 25OHD and 1,25(OH)_2_D, which are converted into more polar and inactive products [13] (Table 2).

From an etiopathogenetic perspective, hypophosphatemic rickets can be subdivided into forms based on reduced phosphate intake or excessive renal phosphate loss, which are associated with hypophosphaturia and hyperphosphaturia, respectively. Nutritional phosphate deficiency rickets results from inadequate intestinal phosphate intake or absorption and is often found in breastfed, extremely preterm babies that are not receiving adequate phosphate supplementation.

Based on renal phosphate loss rate, these forms may be further subdivided into three main groups: (1) hypophosphatemic rickets, attributed to hyperparathyroidism and vitamin D deficiency and which includes acquired or congenital pathological conditions of hypovitaminosis D; (2) FGF23-mediated hypophosphatemic rickets, which includes congenital syndromes and acquired forms; and (3) hypophosphatemic rickets, attributed to non-FGF23-mediated intrinsic kidney defects. In the latter group, acquired or genetic forms are differentiated, but both may resemble renal Fanconi syndrome [14].

Finally, those rickets forms associated with organ disease generally result from a congenital or acquired failure of the organs involved in the regulation of the vitamin D metabolism, i.e., intestine, liver, and kidney (Table 3).

## 6. Clinical Manifestation and Radiologic Features

The classic signs of rickets are related to non-mineralized osteoid tissue buildup in growth cartilages, which is manifested by the appearance of the so called “rachitic cuff” at limb extremities, the typical thorax “rachitic rosary”, which occurs due to the widening of the chondro-costal junctions, and Harrison’s furrow, caused by an indentation of the lower part of the thorax at the point of insertion of the diaphragm muscle. In the lower limbs, as infants start to walk, the femur, tibia, and fibula may bow in varus, but valgus knees or “blow-out” deformities are also possible. Other clinical features include craniotabes, occipital platybasia, craniosynostosis, prominence of the frontal bosses with caput quadratum, and delayed fontanel closure. Scoliosis, and/or dorso-lumbar kyphosis may occur and develop in the spine; in severe cases, deformities of the spine, pelvis, and lower limbs may lead to short stature [15].

In nutritional rickets, delayed tooth eruption (e.g., absence of incisors beyond 10 months and/or absence of molars beyond 18 months of age) or enamel hypoplasia may be evident, resulting in increased susceptibility to caries, even in the permanent teeth.

Extra skeletal manifestations include muscle hypotonia, bone pain [14], increased frequency of respiratory infections, hypochromic anemia, hypocalcemia, which may be asymptomatic, latent (positivity of Chvostek and Trousseau signs), or symptomatic, with acute onset and seizures [16], and dilated cardiomyopathy, that may lead to heart failure or even death [17].

Moreover, in above 51% of patients with X-linked hypophosphatemic (XLH) rickets, dental and periodontal lesions, especially abscesses with gingival fistulas, have been reported, even in the absence of traumas and/or caries. Such lesions are most common in the canine and incisor teeth [18,19]. In adulthood, patients with XLH may develop osteomalacia, enthesopathy, degenerative joint processes, and dental or periodontal changes with recurrent periapical abscesses that may lead to premature edentulousness [20].

Besides the above-mentioned clinical features, radiological signs of rickets are detectable at the level of rapidly growing bones, such as the radius, ulna, distal femur, and proximal and distal tibia. Metaphyseal enlargement, delayed appearance of the ossification centers, osteopenia, cortical thinning of the long bones, osteomalacia, and greenwood fractures are the most commonly found radiological features [12]. 

The Rickets Severity Score (RSS) [21] is a useful scoring method to assess rickets severity by means of bilateral wrist and knee radiographs, based on the degree of fraying and concavity of the metaphysis, and on the proportion of growth cartilage affected (Table 4).

## 7. Diagnostic Approach to Suspected Rickets

If rickets is suspected, the evaluation of specific biochemical parameters, such as serum calcium, phosphate, alkaline phosphatase (ALP), PTH, and vitamin D metabolites, is mandatory for a diagnosis [12].

In all types of rickets, a peculiar biochemical feature, albeit not a pathognomonic, is the presence of increased serum ALP, which is more relevant in calcipenic than in phosphopenic rickets [22]. ALP assessment also helps identify diseases that may mimic rickets, such as hypophosphatasia, which is characterized by low serum ALP concentrations. Decreased ALP activity leads to the accumulation of pyridoxal 5′-phosphate (PLP), inorganic pyrophosphate (PPi), and phosphoethanolamine. Impairment in PLP dephosphorylation induces seizures, while PPi accumulation inhibits bone mineralization. Consequent clinical manifestations may include rickets-like bone changes, bone demineralization, fragility fractures, reduced muscular strength, chest deformity, pulmonary hypoplasia, nephrolithiasis, nephrocalcinosis, and chondrocalcinosis. Treatment relies on enzyme replacement therapy. The use of ALP to determine disease activity has been proven to be useful in monitoringtherapeutic treatment [23].

Serum calcium concentrations may be reduced in cases of severe vitamin D deficiency and in genetically determined vitamin D defective hydroxylation. 

Phosphate concentrations are reduced in hypophosphatemic rickets, as well as in more severe forms of nutritional vitamin D deficiency rickets, where they reflect secondary hyperparathyroidism.

PTH concentrations are increased in forms with hypocalcemia, being normal or just slightly increased in hypophosphatemic rickets; rare exceptions include the X-linked recessive form and the one with hypercalciuria [12,24] (Table 5).

The concentration of 25(OH)vitamin D reflects the body’s vitamin D reserves and is usually decreased in case of deficiency. Recently, there was global consensus on setting 30 ng/mL as the cutoff serum concentration for vitamin D deficiency in nutritional rickets [3]. The risk of rickets increases with decreasing serum vitamin D concentrations and is particularly high below 10 ng/mL, even in the presence of adequate Ca intake. Most children with vitamin D deficiency rickets have serum 25(OH)D concentrations below 10 ng/mL, and usually even below 5 ng/mL [25]. Table 6 shows the definition of Vitamin D status according to different international societies and organizations, as reported in the Italian Consensus document on Vitamin D during the pediatric years [11]. Table 7 shows the most recent definition, as reported by Pludowski et al. in the Polish Guidelines on Vitamin D deficiency prevention and treatment [8].

## 8. Treatment

Rickets therapy is based on vitamin D and calcium supplementation, plus phosphate when necessary. In conditions of malabsorption/malnutrition, combined vitamin D and calcium treatment is mandatory, whereas dietary calcium intake may be sufficient in cases of rickets secondary to metabolic disorders, even though vitamin D supplementation remains fundamental. Treatment with vitamin D in rickets due to calcium deficiency has also been hypothesized. Although the first studies on this topic were based on drugs containing both vitamin D and calcium, due to a lack of evidence, it is conceivable that supplementing vitamin D with calcium may not always be necessary and may also increase the risk of side effects such as nephrocalcinosis and kidney stones [25].

### 8.1. Nutritional Rickets due to Vitamin D Deficiency

Early treatment of this form of rickets is based on the combined administration of vitamin D and calcium salts [26]. Calcium salts dose (30–75 mg/kg, 2–3 times per day) varies according to body weight. In case of symptomatic hypocalcemia, intravenous administration of 5–20 mg/kg of calcium salts (calcium gluconate 10%) every 4–6 h, with careful electrocardiographic (ECG) monitoring, is indicated. The dose of vitamin D to be supplemented is tailored according to the patient’s age [27] (Table 8).

Vitamin D2 or D3 therapy can also be applied as a “one shot therapy”, also referred to as “stoss therapy” (Table 9).

It has been claimed that this approach improves therapeutical compliance in patients who are reluctant to adhere to the daily dose intake, besides being easier to apply. As for potential concerns about the safety of large single doses, hypercalcemia and/or hypercalciuria has been seldom reported as a side effect; in a Turkish study on nutritional rickets, only 8 out of 56 children aged 3–36 months, two of whom were receiving 300,000 IU and six 600,000 IU, developed hypercalcemia [28]. Another study carried out in India, comparing single oral doses of 300,000 vs. 600,000 IU of vitamin D3 in 76 children aged 6 months to 5 years with nutritional rickets, reported the occurrence of hypercalcemia in only five children (two in the 300,000 IU and three in the 600,000 IU group) [29].

With regard to the mode of administration, the oral route should be preferred; indeed, a study carried out on an adult population showed that orally delivered vitamin D led to higher serum 25(OH)vitamin D concentrations after 3 and 6 months compared to intramuscular administration [30]. Though vitamin D2 and D3 have been considered equally active for many years, current knowledge indicates that vitamin D2 efficacy is less than a third of that of vitamin D3 [31]. Studies have also demonstrated that daily vitamin D2 and vitamin D3 intakes are equally effective, whereas vitamin D3 should be recommended in case of a single dose treatment due to its longer half-life. The chemical structures of ergocalciferol and cholecalciferol are similar but not identical; vitamin D3 has a double bond and an additional methyl group on the side chain, and it is supposed that its different structure may identify cholecalciferol as the preferred substrate in different steps of the vitamin D metabolism pathway. There are data suggesting that differences in the side chains of the two forms of vitamin D directly influence the hepatic vitamin D hydroxylation rate, with vitamin D3 thought to be the preferred substrate for hepatic 25-hydroxylase [30]. Vitamin D3 and its metabolites also have a higher affinity to vitamin D binding protein compared to vitamin D2. In addition to these metabolic differences between the two forms of vitamin D, vitamin D3 degradation requires an additional step compared to vitamin D2, suggesting a higher degradation rate for vitamin D2 than for vitamin D3 [24,31].

Vitamin D treatment is recommended for at least 12 weeks, though some children may require longer treatment duration. At the end of the treatment period, maintenance therapy with different doses (400–1000 IU/day) according to age is recommended [32].

### 8.2. Genetic Vitamin-D Dependent Rickets

As for vitamin D-dependent rickets, treatment is based on the combined administration of an active vitamin D metabolite and calcium salts. The most commonly used active metabolites are calcitriol, which regulates the active transport of calcium from the intestine and suppresses the secretion of parathyroid hormone, and alfacalcidiol, which does not require renal activation. Calcitriol has a half-life of approximately 5–8 h; thus, at least 2–3 daily doses are required. In contrast, alfacalcidiol, despite having a shorter efficacy, has a longer half-life (approximately 24 h), allowing a single daily administration. However, their clinical efficacy is overlapping [33].

Treatment of genetic vitamin D-dependent rickets must be continued for life, with patient tailored doses.

The treatment of genetic vitamin-D-dependent tickets type 1B is based on the administration of cholecalciferol, i.e., at different doses, depending on the heterozygous or monozygous mutation status, or of calcifediol [34]. Several studies have shown that oral calcifediol causes a faster increase in serum 25OH-vitamin D compared to oral cholecalciferol, also requiring lower doses [35]. Patients with genetic vitamin D-dependent rickets type 2 without alopecia show a better response to treatment than patients with alopecia [12]. Therapeutic monitoring is very important to control serum calcium, phosphate, ALP, PTH, and urinary calcium excretion (Table 10).

### 8.3. X-Linked Hypophosphatemic Rickets (XLH)

The treatment of FGF23-independent forms of hypophosphatemic rickets, due to genetic defects inhibiting renal tubular phosphate reabsorption, only requires oral phosphate, as these forms are associated with excessive 1,25 (OH)2 vitamin D production. Finally, hypophosphatemic rickets forms caused by Fanconi syndrome, such as nephropathic cystinosis and Dent’s disease, require disease-specific treatment in addition to phosphate supplements of active forms of vitamin D [36,37].

FGF23-associated hypophosphatemic rickets has usually been treated with frequent doses of oral phosphate salts in combination with active forms of vitamin D. Inorganic phosphate salts require multiple administrations throughout the day (4–6 doses) due to their short half-life and to reduce intestinal side effects (e.g., diarrhea, abdominal discomfort) (Table 11).

Patients who show a poor response to conventional treatment or significant side effects are candidates for therapy with Burosumab, a humanized IgG1 monoclonal antibody directed against the FGF23 hormone.

The mechanism of action of this neutralizing antibody is the inactivation of serum FGF23 by enhancing renal phosphate reabsorption and increasing its circulating concentrations. Burosumab treatment is approved in XLH pediatric patients aged between 1 and 17 years and in patients with radiological evidence of bone disease. Burosumab has many advantages over conventional treatment: it increases therapeutic adherence, thereby eliminating the need to take drugs several times a day, and it is not associated with undesirable side-effects, such as gastrointestinal discomfort, diarrhea, secondary hyperpathyroidism, hypercalciuria, or nephrocalcinosis. The recommended dose of Burosumab ranges from 0.8 to 2.0 mg/kg, up to a maximum dose of 90 mg. The drug should be administered subcutaneously every 2 weeks [38].

A randomized, controlled trial performed with children aged 1–12 years, who were randomly selected to receive either Burosumab therapy bi-weekly for 40 weeks or conventional therapy for 40 weeks, showed that the severity of rickets and the body height achieved were significantly improved in the group treated with the monoclonal antibody compared to conventional therapy [39]. Besides this, a phase 2 study performed with children with XLH, aged 1–4 years, showed a significant increase in serum circulating phosphate concentrations [40].

Overall, Burosumab treatment has proved to be more effective than conventional therapy in improving rickets, growth, lower limb deformities, and walking in children with XLH from 1 to 12 years of age.

Some patients with XLH have also been treated with growth hormone (GH) in combination with conventional treatment to improve growth. GH is able to promote phosphate homeostasis by modulating the ratio of tubular maximum reabsorption of phosphate (TmP) to glomerular filtration rate (GFR) (TmP/GFR), but the effects appear to be transient. Patients with XLH, except for a few sporadic cases, do not show GH deficiency in stimulation tests. Therapeutic trials with associated administration of GH have shown inconclusive results [41,42,43]. In conclusion, there are no clinical or biochemical data to predict a possible positive response to GH therapy in XLH patients. Finally, Burosumab represents a second line therapy in adults with XLH rickets with osteomalacia and pseudofractures who are not responding to conventional treatment, or in patients who are intolerant to conventional treatment [38,44,45].

## 9. Conclusions

Vitamin D is crucial for bone development and homeostasis throughout life. Its supplementation, even in the prenatal period, is crucial for the prevention of nutritional rickets; it also plays a key role in the treatment of vitamin D-dependent genetic rickets. Several vitamin D formulations are commercially available for the treatment of the different forms of rickets.

The choice of the form of vitamin D, as well as of an active metabolite, should be made according to the underlying defect in vitamin D metabolism that inhibits its adequate absorption, synthesis, or transport. In cases of vitamin D-dependent genetic rickets, supplementation with metabolites downstream of the genetic defect is strongly recommended to ensure that an adequate phospho–calcium balance is achieved.

In XLH rickets, therapy with the monoclonal antibody Burosumab has shown excellent results in terms of improved growth, increased serum phosphate and vitamin D concentrations, and decreased circulating ALP values, which are reliable markers of disease status. Despite its evident efficacy, Burosumab use is currently hampered by its high cost; moreover, there are no studies to date on its long-term effects or on its effects on disabling conditions in adulthood (e.g., enthesopathies, osteoarthritis, spinal stenosis).

## Figures and Tables

**Table 1:** Rickets prevention by vitamin D supplementation.

Age	Dose IU
0–3 months	400/die
1–3 years	600/die
4–10 years *	600–1000/die
11–18 years *	1000–2000/die
19–65 years *	1000–2000/die
65–75 years	1000–2000/die
>75 years	2000–4000/die

* In persons who sunbathe with bare forearms and legs for 30–45 min between 10 a.m. and 3 p.m. without sunscreen from May until June, supplementation is not necessary, even though it is recommended and safe. In people who do not fulfill these criteria, supplementation is recommended throughout the year [8].

**Table 2:** Causes of calcipenic rickets.

Calcipenic Rickets	Causes
Nutritional rickets	Calcium deficiency
	Vitamin D deficiency
Congenital defects of action of Vitamin D	Vitamin D dependent rickets type 1A Vitamin D dependent rickets type 1B Vitamin D dependent rickets type 2A Vitamin D dependent rickets type 2B Vitamin D dependent rickets type 3
Acquired vitamin D deficiency	Hepatic insufficiency
	Renal insufficiency
	Malabsorption
	Drugs
	Hyperparathyroidism

**Table 3:** Causes of hypophosphatemic rickets.

Hypophosphatemic Rickets	Causes
Nutritional/reduced intestinal intake/absorption of phosphates	Malnutrition
	Prematurity
	Phosphate chelators Administration of elemental formulas Total parental nutrition Gastrointestinal nutrition (e.g., short bowel syndrome) Cellular re-distribution Insulin therapy in diabetic ketoacidosis Acute respiratory alkalosis Re-feeding syndrome
Increased renal phosphate loss	FGF23-related hypophosphatemia
	Hypophosphatemic rickets with hypercalciuria
Renal proximal tubule dysfunction/Fanconi’s syndrome	Oculocerebrorenal syndrome
	X-linked recessive nephrolithiasis
	Infantile cystinosis Tyrosinemia Hepatolenticular degeneration

**Table 4:** Rickets Severity Score (RSS).

	Grade	Description
Wrist ^1^	0	normal growth cartilage without signs of rickets
	0.5	metaphyseal margin radiolucency without enlargement or irregularity of the metaphyseal margin
	1	enlargement of growth cartilage, irregularity of metaphyseal margin
	1.5	partial metaphyseal concavity or incomplete irregularity of metaphyseal margin
	2	concave appearance of the metaphyses with fraying of the margins
Knee ^2^	0	normal growth cartilage without signs of rickets
	1	partial radiolucency, regular margins of metaphyses
	2	partial radiolucency, irregular margins of the metaphyses
	3	complete radiolucency, the epiphyses are clearly separated from metaphyses

^1^ Assessing the ulna and radius individually; maximum score possible: 4. ^2^ Assessing the femur and tibia individually; multiplying the grade for the portion of cartilage affected: 0.5 if <1 condylus affected; 1 if 2 condylus are affected; maximum score 6.

**Table 5:** Biochemical finding in rickets.

	Ca	P	ALP	uCa	FGF23	PTH	25OHD	1,25(OH)2D
Nutritional Rickets with vitamin D deficiency	N, <	N, <	>	<	N, <	N	<	>, N, <
Nutritional Rickets with Ca deficiency	N, <	N, <	>	<	>	N, >	N, <	>
XLH rickets	N	<	>	N	>	N, >	N	N, <
Hereditary hypophosphatemic Rickets with hypercalciuria	N	<	>	>	<	N, <	N	>
Hypophosphatemic Rickets with Hyperparathyroidism	N	<	>	N	>	>	N	N
Vitamin D-dependent R. type 1A	<	<	>	<	N, <	>	N, >	<
Vitamin D-dependent R. type 1B	N, <	<	>	<	?	>	<	<
Vitamin D-dependent R. type 2A	<	<	>	<	N, <	>	N, >	>
Vitamin D-dependent R. type 2B	<	<	>	<	?	>	N, >	>
vitamin D-dependent R. type 3	<	<	>	<	?	>	<	<

N: normal level; >: high level; <: low level; ?: not known.

**Table 6:** Definitions of Vitamin D status worldwide; modified from [11].

Society/Organization	Year	Severe Deficiency	Deficiency	Insufficiency	Sufficiency/ Adequacy
Canadian Pediatric Society	2007	-	<10 ng/mL	10–29 ng/mL	≥30 ng/mL
Lawson Wilkins Pediatric Endocrine Society	2008	<5 ng/mL	5–14 ng/mL	15–19 ng/mL	≥20 ng/mL
Institute of Medicine	2011	-	<12 ng/mL	12–20 ng/mL (a)	≥20 ng/mL
The Endocrine Society	2011	-	<20 ng/mL	21–29 ng/mL	≥30 ng/mL
British Paediatric and Adolescent Bone Group	2012	-	< 10 ng/mL	10–19 ng/mL	≥20 ng/mL
French Society of Paediatrics	2012	-	< 20 ng/mL	-	≥20 ng/mL
Asociación Espanola de Pediatría (Spain)	2012	-	<20 ng/mL	-	≥20 ng/mL
Federal Commission for Nutrition (Switzerland)	2012	<10 ng/mL	<20 ng/mL	-	≥20 ng/mL
Nordic Nutrition Recommendations	2012	-	<12 ng/mL	12–20 ng/mL	≥20 ng/mL
German Nutrition Society	2012	-	-	-	≥20 ng/mL
Health council of the Netherlands	2012	-	-	-	≥12 ng/mL
European Society for Paediatric Gastroenterology Hepatology and Nutrition	2013	<10 ng/mL	<20 ng/mL	-	≥20 ng/mL
Central Europe	2013	-	<20 ng/mL	20–29 ng/mL	≥30 ng/mL
Society for Adolescent Health and Medicine	2013	-	<20 ng/mL	20–29 ng/mL	≥30 ng/mL
Australia/New Zealand	2013	<5 ng/mL	5–11 ng/mL	12–19 ng/mL	≥20 ng/mL
American Academy of Pediatrics	2014	-	<20 ng/mL	-	≥20 ng/mL
Japanese Society for Bone and Mineral Research, Japan Endocrine Society (b)	2015	-	<20 ng/mL	-	-
Scientific Advisory Committee on Nutrition	2016	-	-	-	≥10 ng/mL
European Food Safety Authority	2016	-	-	-	≥20 ng/mL
United Arab Emirates	2016	-	<20 ng/mL	20–29 ng/mL	≥30 ng/mL
Global Consensus for rickets	2016	-	<12 ng/mL	12–19 ng/mL	≥20 ng/mL
Japanese Society for Bone and Mineral Research, Japan Endocrine Society (c)	2017	-	<20 ng/mL	20–29 ng/mL	≥30 ng/mL
European Academy of Pediatrics	2017	Definition of vitamin D status is unclear due to a lack of consensus			

(a) Vitamin D inadequacy; (b) Diagnostic criteria for rickets; (c) Assessment criteria for vitamin D deficiency/insufficiency (Authors reported that different criteria may be needed for children).

**Table 7:** Recent Polish definition of Vitamin D status; modified from [8].

	2009 Polish Recommendations	2013 Central European Recommendations	2018 Polish Recommendations
Diagnostics Thresholds Defining Vitamin D Status on the Basis of Serum 25(OH)D Concentration [ng/mL] *			
Sufficiency	Children: 20–60 Adults: 30–80	30–50	30–50
Insufficiency	Not defined	20–30	20–30
Deficiency	<10	<20	10–20 deficiency <10 severe deficiency
Toxicity	Not defined	>100	>100

* 1 ng/mL = 2.5 nmol/L.

**Table 8:** Therapy of nutritional vitamin D deficiency rickets by daily administration of vitamin D.

Age	Dose IU	Duration
<3 months	2000	3 months
3–12 months	2000	3 months
>1–12 years	3000–6000	3 months
>12 years	6000	3 months

Table adapted from Munns et al. [3].

**Table 9:** Therapy of nutritional vitamin D deficiency rickets by single or repeated vitamin D administration; adapted from Munns et al. [3].

Age	Dose IU	Duration
3–12 months	50,000	Single dose
>1–12 years	150,000	Single dose
>12 years	300,000	Single dose

**Table 10:** Treatment of genetic vitamin-D dependent rickets.

	Vitamin D or Metabolites	Calcium Salt
Vitamin D dependent rickets type 1A	Alfacalcidiol or calcitriol 10–100 ng/kg/day	0.5–3 g/day in 2–3 doses
Vitamin D dependent rickets type 1 B	Homozygous patient: vitamin D 600,000 IU/every 3 months	0.5–2 g/day in 2–3 doses
	Heterozygous patients: vitamin D 5000–10,000 IU/day or	
	Calcifediol 15–50 µg/day	
Vitamin D dependet rickets type 2	Calcitriol or alfacalcidol 10–400 ng/kg/day	3–6 g/day in 2–3 doses
Vitamin D dependent rickets type 3	Vitamin D 20,000–50,000/day	

**Table 11:** Standard treatment for XLH rickets.

	Treatment Before the Development of Clinical or Radiological Signs and Symptoms	Treatment in Presence of Clinical Signs or Symptoms
Start dose	Alfacalcidiol: 25–40 ng/kg/day	Alfacalcidiol: 40–80 ng/kg/day
	Calcitriol: 20–30 ng/kg/day	Calcitriol: 20–40 ng/kg/day
	Inorganic phosphate salts: 40–60 mg/kg/day (divided into 4–6 doses/day)	Inorganic phosphate salts: 40–60 mg/kg/day (divided into 4–6 doses/day)
Retention dose	Alfacalcidiol: 25–40 ng/kg/day (1–2 µg/day) Calcitriol: 20–40 ng/kg/day Inorganic phosphate salts: 30–60 mg/kg/day (divided into 4–6 doses/day)	

## Data Availability

Data are contained within the article.

## References

[1] Chanchlani R., Nemer P., Sinha R., Nemer L., Krishnappa V., Sochett E., Safadi F., Raina R. (2020). An Overview of Rickets in Children. Kidney Int. Rep..

[2] Simm P.J., Munns C.F., Jefferies C.A., Wheeler B.J. (2020). Editorial: Childhood Rickets—New Developments in Epidemiology, Prevention, and Treatment. Front. Endocrinol..

[3] Munns C.F., Shaw N., Kiely M., Specker B.L., Thacher T.D., Ozono K., Michigami T., Tiosano D., Mughal M.Z., Mäkitie O. (2016). Global Consensus Recommendations on Prevention and Management of Nutritional Rickets. J. Clin. Endocrinol. Metab..

[4] Janoušek J., Pilařová V., Macáková K., Nomura A., Veiga-Matos J., Silva D.D.D., Remião F., Saso L., Malá-Ládová K., Malý J. (2022). Vitamin D: Sources, physiological role, biokinetics, deficiency, therapeutic use, toxicity, and overview of analytical methods for detection of vitamin D and its metabolites. Crit. Rev. Clin. Lab. Sci..

[5] Khazai N., Judd S.E., Tangpricha V. (2008). Calcium and vitamin D: Skeletal and extraskeletal health. Curr. Rheumatol. Rep..

[6] Waldron J.L., Ashby H.L., Cornes M.P., Bechervaise J., Razavi C., Thomas O.L., Chugh S., Deshpande S., Ford C., Gama R. (2013). Vitamin D: A negative acute phase reactant. J. Clin. Pathol..

[7] Holick M.F. (2017). The vitamin D deficiency pandemic: Approaches for diagnosis, treatment and prevention. Rev. Endocr. Metab. Disord..

[8] Płudowski P., Kos-Kudła B., Walczak M., Fal A., Zozulińska-Ziółkiewicz D., Sieroszewski P., Peregud-Pogorzelski J., Lauterbach R., Targowski T., Lewiński A. (2023). Guidelines for Preventing and Treating Vitamin D Deficiency: A 2023 Update in Poland. Nutrients.

[9] Thacher T.D., Pludowski P., Shaw N.J., Mughal M.Z., Munns C.F., Högler W. (2016). Nutritional rickets in immigrant and refugee children. Public. Health Rev..

[10] Byrne S.N. (2014). How much sunlight is enough?. Photochem. Photobiol. Sci..

[11] Saggese G., Vierucci F., Prodam F., Cardinale F., Cetin I., Chiappini E., De’ Angelis G.L., Massari M., Miraglia Del Giudice E., Miraglia Del Giudice M. (2018). Vitamin D in pediatric age: Consensus of the Italian Pediatric Society and the Italian Society of Preventive and Social Pediatrics, jointly with the Italian Federation of Pediatricians. Ital. J. Pediatr..

[12] Acar S., Demir K., Shi Y. (2018). Genetic Causes of Rickets. J. Clin. Res. Pediatr. Endocrinol..

[13] Carpenter T.O., Shaw N.J., Portale A.A., Ward L.M., Abrams S.A., Pettifor J.M. (2017). Rickets. Nat. Rev. Dis. Primers.

[14] Roizen J.D., Li D., O’Lear L., Javaid M.K., Shaw N.J., Ebeling P.R., Nguyen H.H., Rodda C.P., Thummel K.E., Thacher T.D. (2018). CYP3A4 mutation causes vitamin D–dependent rickets type 3. J. Clin. Investig..

[15] Mughal M.Z. (2011). Rickets. Curr. Osteoporos. Rep..

[16] Wheeler B.J., Dickson N.P., Houghton L.A., Ward L.M., Taylor B.J. (2015). Incidence and characteristics of vitamin D deficiency rickets in New Zealand children: A New Zealand Paediatric Surveillance Unit study. Aust. N. Z. J. Public. Health.

[17] Basatemur E., Sutcliffe A. (2015). Incidence of Hypocalcemic Seizures Due to Vitamin D Deficiency in Children in the United Kingdom and Ireland. J. Clin. Endocrinol. Metab..

[18] Maiya S., Sullivan I., Allgrove J., Yates R., Malone M., Brain C., Archer N., Mok Q., Daubeney P., Tulloh R. (2008). Hypocalcaemia and vitamin D deficiency: An important, but preventable, cause of life-threatening infant heart failure. Heart.

[19] Baroncelli G.I., Zampollo E., Manca M., Toschi B., Bertelloni S., Michelucci A., Isola A., Bulleri A., Peroni D., Giuca M.R. (2021). Pulp chamber features, prevalence of abscesses, disease severity, and PHEX mutation in X-linked hypophosphatemic rickets. J. Bone Miner Metab.

[20] Chaussain-Miller C., Sinding C., Wolikow M., Lasfargues J.-J., Godeau G., Garabédian M. (2003). Dental abnormalities in patients with familial hypophosphatemic vitamin D-resistant rickets: Prevention by early treatment with 1-hydroxyvitamin D. J. Pediatr..

[21] Thacher T.D., Fischer P.R., Pettifor J.M., Lawson J.O., Manaster B.J., Reading J.C. (2000). Radiographic scoring method for the assessment of the severity of nutritional rickets. J. Trop. Pediatr..

[22] Cannalire G., Pilloni S., Esposito S., Biasucci G., Di Franco A., Street M.E. (2023). Alkaline phosphatase in clinical practice in childhood: Focus on rickets. Front. Endocrinol..

[23] Gentile C., Chiarelli F. (2021). Rickets in Children: An Update. Biomedicines.

[24] Chesher D., Oddy M., Darbar U., Sayal P., Casey A., Ryan A. (2018). Outcome of adult patients with X-linked hypophosphatemia caused by *PHEX* gene mutations. J. Inher. Metab. Dis..

[25] Wilson L.R., Tripkovic L., Hart K.H., Lanham-New S.A. (2017). Vitamin D deficiency as a public health issue: Using vitamin D _2_ or vitamin D _3_ in future fortification strategies. Proc. Nutr. Soc..

[26] Thacher T.D., Fischer P.R., Pettifor J.M. (2014). Vitamin D treatment in calcium-deficiency rickets: A randomised controlled trial. Arch. Dis. Child..

[27] Shaw N.J. (2016). Prevention and treatment of nutritional rickets. J. Steroid Biochem. Mol. Biol..

[28] Misra M., Pacaud D., Petryk A., Collett-Solberg P.F., Kappy M., on behalf of the Drug and Therapeutics Committee of the Lawson Wilkins Pediatric Endocrine Society (2008). Vitamin D Deficiency in Children and Its Management: Review of Current Knowledge and Recommendations. Pediatrics.

[29] Cesur Y., Caksen H., Gündem A., Kirimi E., Odabas D. (2003). Comparison of low and high dose of vitamin D treatment in nutritional vitamin D deficiency rickets. J. Pediatr. Endocrinol. Metab..

[30] Mittal H., Rai S., Shah D., Madhu S.V., Mehrotra G., Malhotra R.K., Gupta P. (2014). 300,000 IU or 600,000 IU of oral vitamin D3fortreatmentofnutritional rickets: A randomized con trolled trial. Indian Pediatr..

[31] Zabihiyeganeh M., Jahed A., Nojomi M. (2013). Treatment of hypovitaminosis D with pharmacologic doses of cholecalciferol, oral *vs* intramuscular; an open labeled RCT. Clin. Endocrinol..

[32] Armas L.A.G., Hollis B.W., Heaney R.P. (2004). Vitamin D _2_ Is Much Less Effective than Vitamin D _3_ in Humans. J. Clin. Endocrinol. Metab..

[33] Wagner C.L., Greer F.R., the Section on Breastfeeding and Committee on Nutrition (2008). Prevention of Rickets and Vitamin D Deficiency in Infants, Children, and Adolescents. Pediatrics.

[34] Levine M.A. (2020). Diagnosis and Management of Vitamin D Dependent Rickets. Front. Pediatr..

[35] Molin A., Wiedemann A., Demers N., Kaufmann M., Do Cao J., Mainard L., Dousset B., Journeau P., Abeguile G., Coudray N. (2017). Vitamin D–Dependent Rickets Type 1B (25-Hydroxylase Deficiency): A Rare Condition or a Misdiagnosed Condition?. J. Bone Miner. Res..

[36] Quesada-Gomez J.M., Bouillon R. (2018). Is calcifediol better than cholecalciferol for vitamin D supplementation?. Osteoporos. Int..

[37] Guidelines of ISPED Study Group on Bone Metabolism and Paediatric Osteoporosis. https://www.siedp.it/files/PDTARachitismiFINALE.pdf.

[38] Haffner D., Leifheit-Nestler M., Grund A., Schnabel D. (2022). Rickets guidance: Part II—Management. Pediatr. Nephrol..

[39] Trombetti A., Al-Daghri N., Brandi M.L., Cannata-Andía J.B., Cavalier E., Chandran M., Chaussain C., Cipullo L., Cooper C., Haffner D. (2022). Interdisciplinary management of FGF23-related phosphate wasting syndromes: A Consensus Statement on the evaluation, diagnosis and care of patients with X-linked hypophosphataemia. Nat. Rev. Endocrinol..

[40] Ward L.M., Glorieux F.H., Whyte M.P., Munns C.F., Portale A.A., Högler W., Simmons J.H., Gottesman G.S., Padidela R., Namba N. (2022). Effect of Burosumab Compared with Conventional Therapy on Younger vs Older Children with X-linked Hypophosphatemia. J. Clin. Endocrinol. Metab..

[41] Imel E.A., Glorieux F.H., Whyte M.P., Munns C.F., Ward L.M., Nilsson O., Simmons J.H., Padidela R., Namba N., Cheong H.I. (2019). Burosumab versus conventional therapy in children with X-linked hypophosphataemia: A randomised, active-controlled, open-label, phase 3 trial. Lancet.

[42] Whyte M.P., Carpenter T.O., Gottesman G.S., Mao M., Skrinar A., San Martin J., Imel E.A. (2019). Efficacy and safety of burosumab in children aged 1–4 years with X-linked hypophosphataemia: A multicentre, open-label, phase 2 trial. Lancet Diabetes Endocrinol..

[43] Haffner D., Emma F., Eastwood D.M., Duplan M.B., Bacchetta J., Schnabel D., Wicart P., Bockenhauer D., Santos F., Levtchenko E. (2019). Clinical practice recommendations for the diagnosis and management of X-linked hypophosphataemia. Nat. Rev. Nephrol..

[44] Linglart A., Biosse-Duplan M., Briot K., Chaussain C., Esterle L., Guillaume-Czitrom S., Kamenicky P., Nevoux J., Prié D., Rothenbuhler A. (2014). Therapeutic management of hypophosphatemic rickets from infancy to adulthood. Endocr. Connect..

[45] Brandi M.L., Jan de Beur S., Briot K., Carpenter T., Cheong H.I., Cohen-Solal M., Crowley R.K., Eastell R., Imanishi Y., Imel E.A. (2022). Efficacy of Burosumab in Adults with X-linked Hypophosphatemia (XLH): A Post Hoc Subgroup Analysis of a Randomized Double-Blind Placebo-Controlled Phase 3 Study. Calcif. Tissue Int..

